# Predicting Attack Pattern via Machine Learning by Exploiting Stateful Firewall as Virtual Network Function in an SDN Network

**DOI:** 10.3390/s22030709

**Published:** 2022-01-18

**Authors:** Senthil Prabakaran, Ramalakshmi Ramar, Irshad Hussain, Balasubramanian Prabhu Kavin, Sultan S. Alshamrani, Ahmed Saeed AlGhamdi, Abdullah Alshehri

**Affiliations:** 1 Department of Computer Science and Engineering, Karpagam College of Engineering, Coimbatore 641032, Tamil Nadu, India; joinwithsenthil@gmail.com; 2 Department of Computer Science and Engineering, Kalasalingam Academy of Research and Education, Krishnankoil 626126, Tamil Nadu, India; rama@klu.ac.in; 3 Faculty of Electrical and Computer Engineering, University of Engineering and Technology, Peshawar 25000, Pakistan; 4 Sri Ramachandra Faculty of Engineering and Technology, Sri Ramachandra Institute of Higher Education and Research, Porur, Chennai 600116, Tamil Nadu, India; prabhukavin@sret.edu.in; 5 Department of Information Technology, College of Computer and Information Technology, Taif University, P.O. Box 11099, Taif 21944, Saudi Arabia; susamash@tu.edu.sa; 6 Department of Computer Engineering, College of Computer and Information Technology, Taif University, P.O. Box 11099, Taif 21944, Saudi Arabia; asjannah@tu.edu.sa; 7 Department of Information Technology, Al Baha University, P.O. Box 1988, Al Baha 65431, Saudi Arabia; aashehri@bu.edu.sa

**Keywords:** software defined network, network function virtualization, firewall, SDNFV, attack prediction, machine learning, decision table, bayesian network

## Abstract

Decoupled data and control planes in Software Defined Networks (SDN) allow them to handle an increasing number of threats by limiting harmful network links at the switching stage. As storage, high-end servers, and network devices, Network Function Virtualization (NFV) is designed to replace purpose-built network elements with VNFs (Virtualized Network Functions). A Software Defined Network Function Virtualization (SDNFV) network is designed in this paper to boost network performance. Stateful firewall services are deployed as VNFs in the SDN network in this article to offer security and boost network scalability. The SDN controller’s role is to develop a set of guidelines and rules to avoid hazardous network connectivity. Intruder assaults that employ numerous socket addresses cannot be adequately protected by these strategies. Machine learning algorithms are trained using traditional network threat intelligence data to identify potentially malicious linkages and probable attack targets. Based on conventional network data (DT), Bayesian Network (BayesNet), Naive-Bayes, C4.5, and Decision Table (DT) algorithms are used to predict the target host that will be attacked. The experimental results shows that the Bayesian Network algorithm achieved an average prediction accuracy of 92.87%, Native–Bayes Algorithm achieved an average prediction accuracy of 87.81%, C4.5 Algorithm achieved an average prediction accuracy of 84.92%, and the Decision Tree algorithm achieved an average prediction accuracy of 83.18%. There were 451 k login attempts from 178 different countries, with over 70 k source IP addresses and 40 k source port addresses recorded in a large dataset from nine honeypot servers.

## 1. Introduction

In conventional networks, network elements are tightly coupled with both data and control planes. There is always one control plane for one data plane in any network device. The management plane acts as an interface to monitor and manage the activities of the network. The huge rise in the amount of Internet-connected systems has led to quite a range of beneficial methods in various areas such as cultivation, medical services, business, trade. The conventional network architectures have been questioned by such an enormous rise in bandwidth demand. In order to meet the demands, the network data plane and control plane is decoupled in Software Defined Network (SDN) [1,2].

### 1.1. SDN

**Figure 1:**
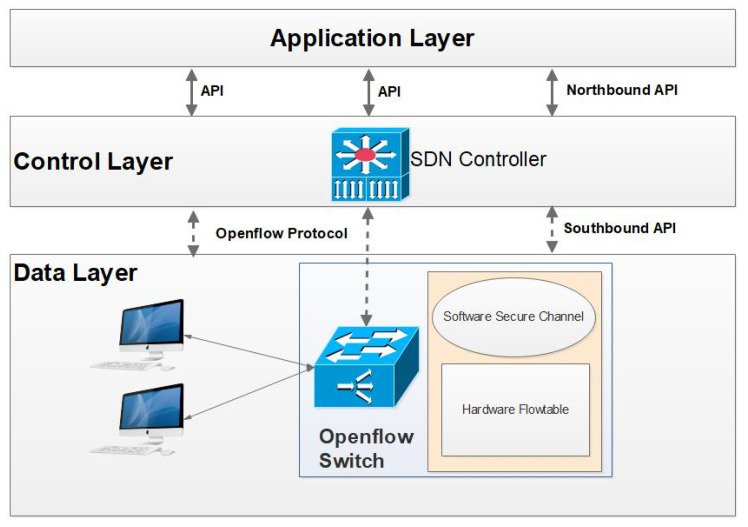
SDN Architecture.

In Software Defined Networks (SDN), network management is made very simple by decoupling the control plane, and data plane form network elements, where the control plane is centralized as a directly programmable controller and the network elements take care of packet forwarding operations. The flexibility of the network is improved because the administrator can configure dynamic rules and policies accordingly. Figure 1 shows the SDN Architecture. SDN facilitates the application to program the network operations through the open northbound API, OpenFlow protocol acts as the southbound API between the controller and forwarding elements.

SDN adapters can be utilized to connect an SDN controller with a legacy network. The key components of the SDN controller and switch interface are OVS switch kernel module, ovs-vswitched daemon with operates over kernel module, and OpenFlow protocol as southbound API. The control agent present in the forwarding element processes all control instructions from the controller using OpenFlow protocol through the southbound API. OVS kernel module manages and maintains the switch forwarding table. The forwarding table is configured by the SDN controller based on functional and management policies. Both synchronous and asynchronous communication takes place in an SDN environment with the support of OpenFlow protocol. SDN eliminates the need for purpose-built dedicated hardware elements with software switches, which run on top of general-purpose hardware to reduce the infrastructural cost [3].

### 1.2. SDN

The service providers are very much in need of new innovative network technologies to overcome the limitations in traditional proprietary hardware and its application. It is a difficult task for an administrator to find space and power to add new network function services and applications in the legacy network data center. NFV almost removes and replaces the requirement to install new services, in turn this drastically reduces the capital and operational expenditure for the service providers. NFV as IT network virtualization technology is emerging as an overall better alternative solution for purpose-built hardware. NFV decouples and virtualizes network functions from physical hardware elements and runs on top of a virtual machine. In NFV, network services like firewall, NAT, DHCP, DNS, IDS, DPI, and so on are separated and converted as a software instance from its purpose-built hardware instance.

**Figure 2:**
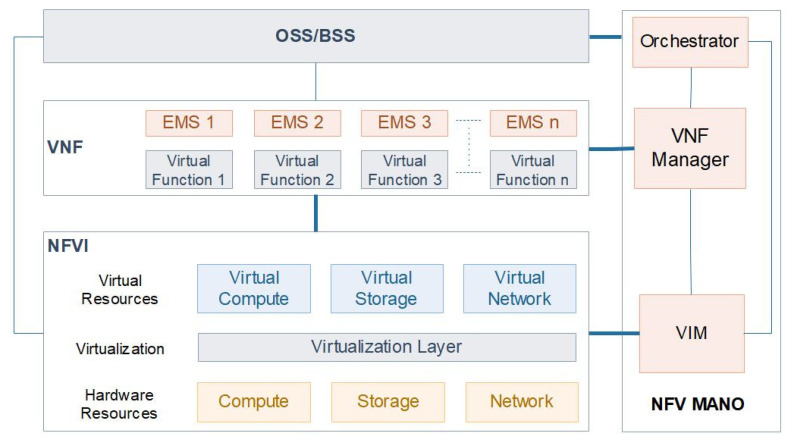
NFV Architecture.

NFV simplifies the network infrastructure services by consolidating all the virtual machines into a single data center server to form a fully virtualized infrastructure. Network services are separated and configured as Virtual Network Functions (VNFs), which can be programmed as software on physical servers. NFV MANO is NFV Management and Orchestration, in which the MANO control flows focus mainly on the management and organization of virtual networks. The main concept of NFV MANO is to allow the adaptable installation and configuration of network elements in a data center and also to avoid network complexity that is related to network availability. Figure 2 shows the NFV Architecture.

NFV Orchestrator, VNF Manager, and Virtualized Infrastructure Manager (VIM) are the major functional blocks of NFV MANO. NFV Orchestrator takes care of deploying virtual network functions (VNFs) and Network Function Virtualized Infrastructure (NFVI) by managing, controlling, authorizing, and administrating the network services and resources. VNF Manager is responsible for VNF components management, where new network services are configured and managed as VNFs. VIM synchronizes the virtual components with hardware components to dynamically deliver and manage network services. As a whole, NFV is a software virtualized platform, which is responsible for deploying network services over purpose-built hardware elements [4]. NFV eliminates various problems such as business expenditure and network complexity by reducing the processing and response time of the network [5].

The control plane programmability is focused by SDN, and the data plane abstraction and virtualization programmability are focused by NFV [6]. With SDN, the complexity of the network has been eliminated without affecting core manual configuration [7]. NFV acts as a facilitator for SDN solutions such as network programmability and centralized control to build a highly available, dynamically configurable, secure, robust, and flexible infrastructure [8]. Such an architecture has the benefit of improving the average performance of the network and enabling improved network management [2]. An intruder can sometimes carry out brute force assaults on the SDN controller, such as a Secure Shell (SSH) attack, which may lead to severe potential risks.

The process of identifying the firewall policies and denying attackers is a complex task. It is observed that attackers commonly use methods such as targeted assaults and password index exchanging [9]. Various machine learning methods can be used to define such behaviors. Machine learning techniques have proved extensive user classification potential [10]. Four distinct machine learning algorithms (BayesNet (BN), Naive-Bayes (NB), Decision Table (DT) and C4.5) are used for predicting susceptible network access, using traditional network attack data. The target host for the attacker is predicted by using AWS honeypot attack data [11] to train different Machine Learning (ML) modules. Using the results of the ML-algorithm, traffic flow rules are identified on the SDN controller by preventing the full sub-network from limiting the entry of future attackers.

This paper’s primary contributions are:

- To deploy a virtual stateful Firewall service to create a firewall VNF component in SDNFV network using the mininet network emulator.
- To define security rules on the stateful firewall VNF for all the traffic flows in and out of the SDNFV network.
- To configure filtering rules in stateful firewall VNF as suggested by the machine learning algorithms to deny access to the target host.
- The efficiency of four commonly used ML algorithms is compared and evaluated. Furthermore, the objective is not to highlight the four algorithms used, but to display the effectiveness of the ML method in SDN security.
• This work proves that even a tiny likelihood of attack acquired via ML strategy has an important impact on the security of the SDN.

This research would be the first to use the Machine Learning method on SDN to define security rules for traffic flows on the stateful firewall VNF to the finest of our understanding. The remaining work is ordered from Section 2, Section 3, Section 4, Section 5 and Section 6 as related works, proposed work, experimental setup, results and discussion and conclusion, respectively.

## 2. Background and Related Work

This section presents a short review of related works on SDN, NFV networks, Methods for machine learning that can be used in Software Defined Networks (SDNs), to manage attack and Distributed Denial of Service (DDoS) threats. Purpose-built and common hardware elements are configured to co-exist on a network to categorize multicast packets and improve network scalability and network reliability [12]. Sudden migration from current traditional networks to SDN and NFV networks is practically difficult to process due to lack of adaptability and flexibility in the current network. Rather, it is better to take a step-by-step migration process [13]. Network services are configured as a software program in hardware element as VNFs, physical hardware resources for VNFs can be configured and migrated dynamically over a multi-domain network [14]. A novel cooperative VNE algorithm is built to organize and synchronize the controller to manage and control the network elements effectively [15]. An SDN architecture is built for virtual tenant networks to dynamically allocate and deploy the available resources by using dynamic routing and bandwidth allocation services [16]. By integrating both SDN and NFV architectures, the performance of the combined architecture is monitored and analyzed using the M/M/1 queuing technique. The performance shows that NFV AC architecture performs better comparing NFV C architecture [17]. To offer better service chaining for network function, a new SDN NFV combined architecture is constructed, and the total capital and operational expenditure are minimized [18]. JANO is introduced in the management plane to dynamically configure and manage virtual network functions, which in turn decrease the time taken by the administrator to report the network-related problems and reduce the control traffic [19].

Multipath SDN network flows are configured with NFV to efficiently control and manage the data plane elements to provide better network performance based on traffic path computation, resource maintenance, periodical forwarding table update and network monitoring [20]. Virtual network Resource (VNR) and Quality of experience (QOE) are two major factors taken into account for improved multimedia applications performance in multimedia aware virtual resource management (MAREM) SDN framework [21]. To resolve problems in linear programming, primal dual algorithm, and heapsort algorithms are used to build a clustered virtual network (CVN). The controller abstracts required physical resources from a single pool or from multiple resource pools as per resource requirements for constructing CVN or cross pod CVN [22]. SDN architecture with residential NFV services is constructed by creating and providing access for Residential Network Management apps (RENEMA apps) at end-user premises. Core level information is screened to build an open architecture for users to manage their own network and facilitate services providers with local network data for better solutions [23]. SDN and NFV technological ideas are utilized to create an Adaptive Routing Service Customization (ARSC) algorithm to dynamically provide services for application-specific routing tasks and resource allocation [24]. Performance analysis and comparison are made for Application-Based Network Operations (ABNO) and single controller orchestration in the SDN cloud environment. The comparison for end-to-end connectivity proves that the network performance is efficient in ABNO SDN model [25]. In the SDN NFV network, a linear programming-based algorithm is proposed for load balancing, baseline, and aggregation to eliminate VNF placement problem [26].

NFV with Software Defined Optical Network is proposed to minimize the overall network delay. It is also observed that bandwidth is inversely proportional to network latency [27]. The network performance is improved with centralized control and automation features in SDN network, which is configured with middle-box functions such as firewall and Intrusion Prevention System (IPS) [28]. SDN and NFV deployed in a cloud environment provide cloud service personalization and minimize latency for end-users and service providers [29]. SDN networks utilize the NFV features for improved network automation, operations, flexibility, fault tolerance, and cost-effectiveness during the process of policy update, vm migration, and other network changes [30]. SDN in distributed cloud datacenter is facilitated with Optimized Virtual Network Provisioning (OVNP) paradigm reduces overall operational expenditure [31]. A performance monitoring architecture for NFV is implemented to measure and analyze the network performance of single and multi-domain virtual network functions [32].

The controller placement algorithm proposed in [33] efficiently handles the virtual node mapping and node links synchronization. To achieve enhancement in network performance with minimized delay and improved throughput, SDN and NFV architectures are combined to achieve centralized control on VNFs [34]. QoS aware virtualization enabled routing (QVR) is implemented to virtualize the routing process in SDN to accomplish less network delay [35]. Bandwidth Risk Ratio algorithm processes vSDN components based on flexibility factor, available bandwidth, and fault tolerance to maximize profit and meet guaranteed SLA policies [36]. An in-depth systematic literature review is conducted on NVF and SDN integrated architecture in view of studying the characteristics, design, tools, SDN APIs, and element placement to build state-of-the-art NFV/SDN architecture, which is open for the future improvements [37]. A network traffic monitoring model for SDN with NFV is designed at reduced costs with improved network management and agility [38]. The performance of TCP and UDP flows in SDN networks are foreseen with two analytical network models by predicting in the advent of both flow level and packet level. It is found that the performance of TCP is much better than the UDP model due to the increased number of packets in UDP model [39]. An SDN network security survey is conducted based on attack identification, traffic engineering, and monitoring, network policy management and configuration, service chaining, and middle-box deployment with smart grid network infrastructure security [40].

A thorough study on the use of SDN to protect networks and recommend the use of SDN as a system for security [41]. The study lists a range of distinct problems and alternatives suggested to account for network risks in the literature. An SDN-focused study on programmable networks [42]. The paper discusses the growth of dynamic networks and shows the design of the SDN network. OpenFlow SDN study and outline OpenFlow’s fundamental ideas, apps, and secure elements [43].

An assessment of the brute force attacks of the automated SSH [44]. The paper used the AWS honeypot attack data to evaluate accurately the activity of the attacker and the attack dynamics, including password dictionary exchanging and targeted attacks. Various SDN anomaly detection methods such as Bayesian Networks, Expectation Maximization, k-Nearest Neighbours (kNN), and Ma-chines Support Vector [45]. In terms of SDN apps, the author discusses various attack situations and their execution. An advanced structure known as Atlas, which leverages SDN application understanding and is suitable for policy implementation based on L2/3/4 [10].

Atlas utilizes the C5.0 classifier for categorizing the flows in SDN, and collecting real-time information using a user-sourcing strategy to incorporate with SDN’s information reporting system’s centralized command. Their suggested scheme is capable of identifying a fine-granular mobile app and achieving a 94% median precision for the top 40 android apps. A general overview on SDN with a specific focus on the current problems in network setup and control processes and suggest various methods for improving network [46].

Flow N has the ability to provide their clients with personal address space, configuration, and control logic. They do use databases to scale processing across physical and virtual networks [47]. A strategy is suggested, as it has distinctive characteristics such as large bandwidth for a fair proportion of data centers, dynamic demand for traffic aimed at maximizing average bandwidth, and rigorous edge server monitoring [48]. Because of the following features, B4 optimizes SDNs enhanced network switch control resulting in nearly 100% use of links.

### 2.1. Machine Learning Techniques

These algorithms are commonly used in Machine learning to deliver precise outcomes for a wide range of classification and prediction issues [49]. Below commonly used ML algorithms defined.

#### 2.1.1. Bayesian Network

Bayes Net computes probabilistic interactions between various interest variables [2]. It comprises a number of factors and a set of vertices, leading to a graph with acyclic variations [50]. The random variable and its directional edge for each node are reflected in the resultant graph. All the variables are autonomous of the non-descendants in the Bayesian network. Bayes Net was used as a classifier and can lead to extremely precise categories if correctly educated. A comprehensive study can be discovered in [51,52,53] on the Bayesian Network.

#### 2.1.2. Native–Bayes

Naive–Bayes utilizes Bayesian theory that employs the training samples to predict the sort of unidentified samples based on previous results. The model of Bayesian classification is based on statistical assessment and the concept of Bayesian, formed for Bayesian learning [54,55]. Bayesian learning combines the preceding and subsequent probability and utilizes it to determine the later probability according to the information and data samples provided. The working of the Native Bayesian algorithm is based on dividing the instruction set as a choice vector and attribute vector. Whereas the algorithm helps each component of the attribute vector by applying the choice variables. A comprehensive discussion can be noticed in [56] on Naive–Bayes.

#### 2.1.3. C4.5

The initial inference process of C4.5 utilizes the decision-making tree in common [57]. The quantified discrete function in C4.5 is used by using a decision tree to reply to the learnt function. Hill climbing heuristic technique is used by C4.5 for searches without a backtracking process on every feasible decision tree. Subgroups information are repeatedly separated from the data acquired. C4.5 is used to learn different phrases of discontinuity, as it is capable of handling extremely noisy data.

#### 2.1.4. Decision Table

The simple initiation process is facilitated by (DT) Decision Table by recording and consolidating the data logic [58]. The machine learning outcomes are used as input data for DT [59], choosing some of the characteristics of the data. They also help to assess various kinds of uncertainty and redundancy rules [60]. Comprehensive discussion can be found outside the range of this document in [58,59,60].

As mentioned in [57], C4.5 DT’s primary learning phases were:

- It selects an attribute modeled on which to evaluate a logical check.
- A Part of training data for the chile node is extracted from the experimental results.
- All the child nodes run the methods iteratively.
- A node is represented by a leaf depending on specific concluding principles.

All the works in SDN/NFV network conducted before are about the traditional network to SDN/NFV network migration process, improving scalability and reliability, dynamic resource allocation, dynamic configuration and management of VNF, VNF service chaining, and migration of VNFs. None of the studies has addressed using machine learning methods to define security rules of virtual stateful firewall VNF as service in SDN/NFV network. This inspired the study.

## 3. Proposed Work

**Figure 3:**
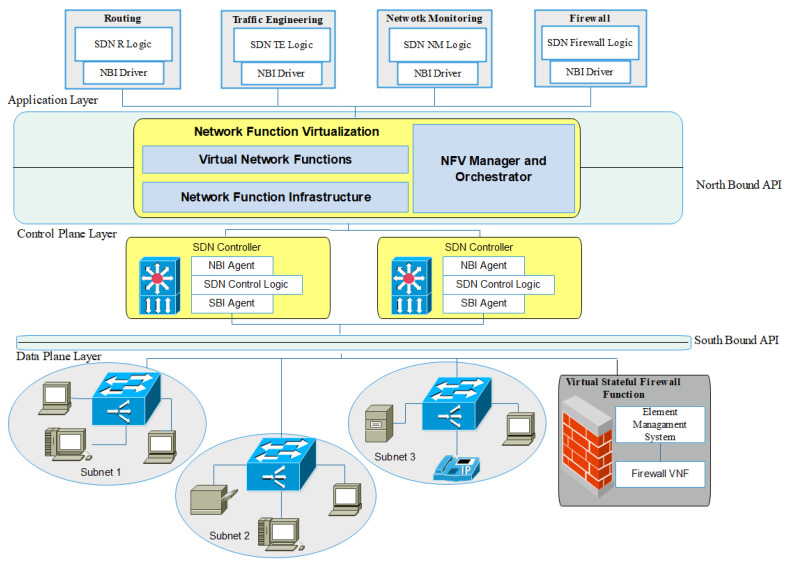
SDNFV with Virtual Stateful Firewall Function.

In this study, the SDN and NFV technologies were combined, and the proposed network was named Software Defined Network Function Virtualization (SDNFV). Figure 3 shows the Proposed SDNFV network with virtual stateful firewall VNF. This work focused on combining the benefits of both SDN and NFV technologies to create a secured SDNFV network with a stateful firewall function.

The proposed stateful firewall was configured to effectively detect and prevent Denial of Service (DoS) Attacks. When there is a large internet security crisis, it is almost always the result of a DDoS assault. These fraudsters frequently attack websites, personal accounts, servers, and other services in order to overwhelm their internet traffic, causing the victim’s system to become indifferent to genuine requests. A denial-of-service (DoS) attack is a sort of computer security threat in which an attacker attempts to make a computer or other network unavailable to authorized users by temporarily or permanently disrupting the regular operations of a host connected to the Internet. A denial-of-service (DoS) attack can assault individual machines or entire computer infrastructure. These assaults may be expensive for a firm until their services, and other impacted resources are restored. The more information available about inbound traffic, the easier it is to detect an attack. Almost all DDoS assaults start with large traffic surges. All of these symptoms might be the result of hackers doing dry runs to test your defenses before launching a full-scale attack. As a result, it would be necessary to distinguish between a sudden spike of real visitors and the start of a DDoS attack. The stateful firewall is used to prevent DOS attacks by tracking and analyzing the status of active network connections while evaluating incoming traffic for possible traffic and data hazards.

Distributed SDN controllers are programmed to control and manage the network with three subnets, and NFV virtual stateful firewall function acts as a gateway firewall for the network. In this design, SDN facilitates NFV ideas to construct a stateful Firewall controlled SDN network with three subnets. The virtualized firewall function is controlled by the distributed SDN controllers. SDN and NFV counterparts to each other very well to build a cost-effective, flexible, and secure network. NFV decouples the network functions to form a physical network element to create VNFs, and SDN separates the control part from the forwarding elements. SDNFV architecture has three-layer, Data layer, Control layer, Application layer, and two application programming interfaces. Southbound API and northbound API are the available APIs where NFV is configured on top of the northbound API. The Control layer and data layer communicate through the southbound interface, and it supports the controller to interact with the network elements and virtual stateful firewall function using OpenFlow protocol. The application layer and control layer interacts through the northbound API. The application layer is configured with basic network function applications such as routing, traffic engineering, network monitoring, and firewall. NFV, which works on top of northbound API as the NFVI component, virtualizes the physical resources to create VNF that is managed by Virtual Infrastructure Manager (VIM). VNFs are created with the virtual compute, storage, and network resources, which are managed by their respective Element Management System (EMS). All the EMSs are controlled and managed by VNF manager. NFV Orchestrator is an overall management interface used to configure and maintain the components of NFV, such as VIM, VNF manager.

In this paper, an SDN network is divided into three subnets to basically prevent packet snooping problems. A virtual stateful firewall function is created and configured with a set of firewall rules and acts as a gateway for the network. Machine learning algorithms are used to detect possible target server threats depending on SDN’s traditional network intrusion data. Four distinct algorithms are used: BayesNet, Naive-Bayes, C4.5, DT, and [61] to predict the victim being targeted and to evaluate their accuracy results. A stateful firewall is deployed in distributed SDN controllers, where the OpenFlow protocol is used by the controllers and switches to send and receive control messages [62,63,64]. IP aliases are created on Stateful Firewall VNF and are updated based on machine learning threat detection algorithms. This way, the traffic flows are matched and allowed in and out of the network, and the rest of the traffic is denied. Virtual Firewall VNF service acts as middlebox between SDN controller and network elements, where all the incoming and outgoing packets are controlled by the controllers. SDN controllers instruct, control, and manage the Stateful Firewall VNF using OpenFlow protocol through the southbound API. The whole SDNFV network setup is deployed in a mini-net emulation environment. POX is an open-source controller configured to handle OpenFlow traffic from the southbound interface and application traffic flows from the northbound interface. After configuring an SDN network, the NFV integration process is initiated. SDN helps NFV to eliminate the need for manual configuration of traffic forwarding on switches and routers, which results in reduced service deployment time.

### 3.1. Function of NFV in SDNFV Network

**Figure 4:**
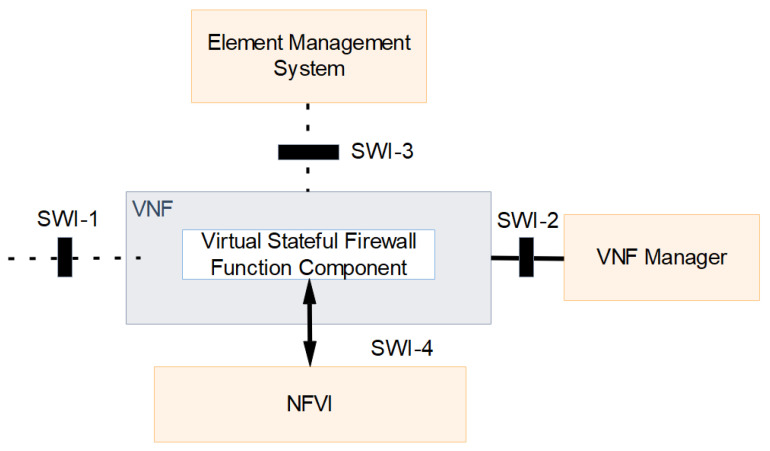
Stateful Firewall VNF interfaces.

NFV comfortably fits using virtualization technology and facilitates the existing network without affecting its operations. This is achieved in NFV by using the server resource and specific control traffic to build virtualized environment. NFV orchestrator provides a high degree resource management service and takes the responsibility of aggregating the compute, storage, and network resources to create a virtual environment that works in parallel with the existing SDN network. Network Firewall function is programmed in a VNF component as a virtual stateful firewall, which is managed by synchronizing element management and VNF Manager Operations. Figure 4 shows the Stateful Firewall VNF interfaces.

Switch Interfaces (SWI) are used for active interaction and information exchange. SWI-1 is the interface used for control and forwarding element interaction, as it links the virtual firewall function with client nodes. SWI-2 interface is used to exchange lifecycle management information with VNF managers, and it is configured as a network link with specific IP address. SWI-3 interface is used for EMS and stateful firewall VNF communication to keep track of VNF runtime information. SW-4 interface communicates with NVFI to allocate resources for a VM container to deploy a stateful firewall VNF component. Service coordination and instantiation process are handled by the orchestrator by communicating with core NFV platform to create a virtual instance of firewall service on the NFV platform. Service chaining is used to scale the created stateful firewall service for the configured network setup. Scaling services are utilized to manage the configured service when the size of the network scales up. The service monitoring function is used to monitor the performance of the resources and configure the service to have stable network operations.

NFV orchestrator management features include Network service life cycle management, resource management, creating new network services, and VNF and NFVI resource request authentication and approval. VIM handles the management of NFVI compute, storage, and network resources, and it helps to interact efficiently with the SDN network elements. VIM function has the network activities tracking feature to maintain and optimize the allotment, upgrade, release, and reclamation operations. VNF function forwarding details are maintained by unifying the virtual function, virtual links, subnets, and ports. As per VNF requirements, the compute, storage, and network resources are dynamically allocated and managed by VIM function. VNF components are controlled and managed by VNF manager. VNF manager supports the virtual network function to maintain standard interaction between SDN and firewall VNF elements. VNF manager has four operations starting with VNF Incorporation, VNF scaling, VNF upgrade, and VNF termination. NFV is utilized to deploy a firewall VNF service in place of a physical hardware firewall service to enhance the scalability and flexibility of the proposed SDNFV network. The stateful filtering rule is configured on the firewall VNF. All traffic packets to and from the network should pass through rules created on a virtual stateful firewall gateway. A firewall rule is created for ingress and out-gress traffic packets. An alias is created with a set of intra network IP addresses and added to the firewall allow rule, and the default rule is added as all remaining IP addresses are blocked.

### 3.2. Machine Learning Framework to Define Stateful Firewall Rules

**Figure 5:**
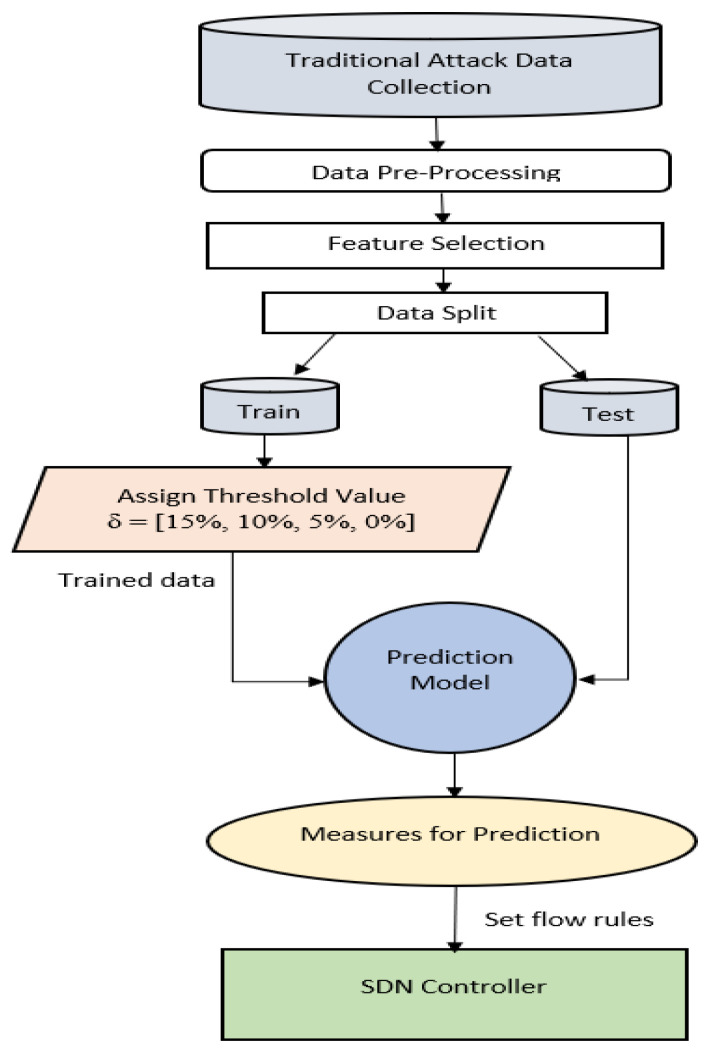
Framework focused on machine learning to define Stateful firewall security rules.

Figure 5 displays the framework based on ML to define SDN controller flow rules.

To train a model, the traditional dataset was used. The trained framework was further used with real-time network data to predict threats on particular hosts accurately, and appropriate rules were defined on the Stateful firewall VNF to prevent the future potential intruder. The core features of the proposed methodology were

1. The use of traditional data to train ML-based applications, and
2. The use of the trained design to detect possibly susceptible hosts and establish the safety rules in the VNF module according to the predictive performance of the Machine learning algorithm. Details are addressed below.

#### 3.2.1. Train ML-Based Designs Using Traditional Data

To train traditional data, machine learning models were used in order to achieve precise classifiers to define possible susceptible hosts. The training assisted the model in learning and achieving better outcomes. The objective utilized the model of traditional attack data to define the possible host that an intruder can attack. A possible host was anticipated, which can be breached based on intruder IP and port addresses. These results can be used to identify SDN module policy rules to determine the safety of the network. Instead of preventing entry to a single IP and Port address, the full subnet sockets were suggested to narrow in order to avoid potential attempts by the same attacker, entering in the same subnet over a distinct IP and port.

#### 3.2.2. Use the Trained Design to Determine Susceptible Host

It is used only after the system is trained to detect possible hosts, which an IP and Port can attack. Trained design was used during the testing stage to identify the targeted host depending on the IP and Port socket of the intruder. If, as expected by the ML algorithm, the intruder effectively violated a host, it states the model is valid and accurate. Model accuracy is determined with Equation (1).

(1) $$\mathrm{Accuracy}=\frac{\mathrm{Number}\;\mathrm{of}\;\mathrm{attacks}\;\mathrm{correctly}\;\mathrm{predicted}}{\mathrm{Number}\;\mathrm{of}\;\mathrm{attacks}\;\mathrm{in}\;\mathrm{total}}*100$$

A threshold level δ percentage is selected as the lowest possible probability needed for any host to be considered vulnerable during testing. The δ values are changed to assess its impact on the accuracy of the ranking. Algorithm 1 describes the proposed method.
**Algorithm 1** Predictor for SDN network attacks modelled on machine learningStep 1: Start Step 2: **Procedure** SDN ATTACK PREDICTOR BASED ON MACHINE LEARNING Step 3: Select algorithm for machine learning Step 4: Train the Machine Learning algorithm with traditional data Step 5: **If** the trained framework predicts an IP and Port Socket hit on a host **then** Step 6:     Update the policies on stateful firewall VNF to restrict the Socket of subnet Step 7: **else** Enable IP and Port Socket access in to network Step 8: End

## 4. Experimental Setup

The proposed SDNFV network was designed using a POX SDN controller programmed with python programming language. The SDNFV network has 3 sub networks, namely subnet 1, subnet 2, and subnet 3. Each sub network has an OpenFlow switch. subnet 1 had 3 host machines, subnet 2 had 2 host machines with a network printer, subnet 3 had two host machines with a VOIP device, and an NFV Virtual firewall VNF service was configured as a gateway firewall for all 3-sub networks. Mininet was used as a network emulation tool with Xming application as display server and putty as a remote client application. The virtual stateful firewall VNF service enabled SDNFV network was configured, deployed, and monitored. The emulation setup is shown in Table 1.

**Figure 6:**
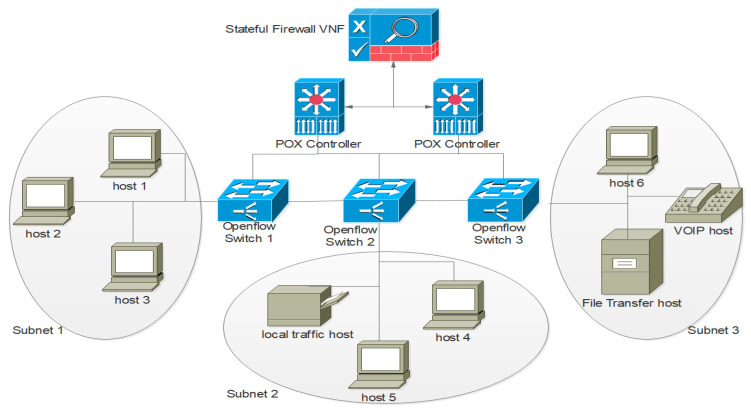
SDNFV Network Topology.

Figure 6 shows the network topology of implemented SDNFV with stateful firewall VNF services. There are three subnetworks as subnet 1, subnet 2, and subnet 3 each subnet has three host machines and respective OpenFlow switches. All the OpenFlow switches are connected to the stateful firewall VNF and POX controller.

**Figure 7:**
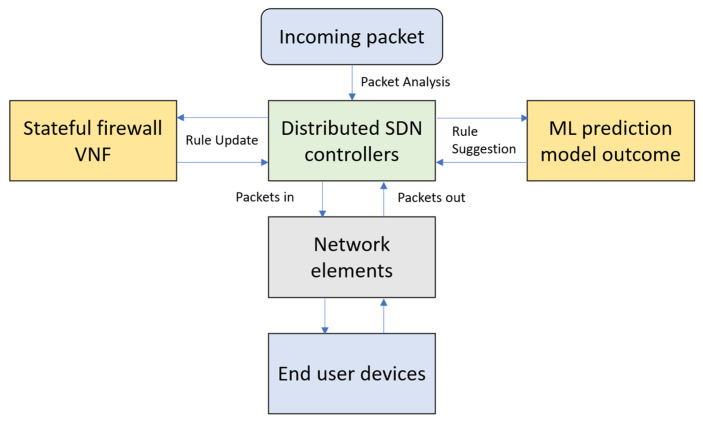
Flow of proposed SDNFV Network.

Figure 7 shows the flow of the proposed SDNFV. When incoming packets arrive in the SDNFV environment, packets are directly forwarded to the controllers, which is accompanied by a stateful firewall inspection module. The stateful firewall module rules are configured based on the prediction suggestion from the machine learning algorithm analysis to predict the DOS attacks based on the training/testing model form the AWS honeypot dataset. Based on the configured firewall packet filtration policies, the packets and their current connection status were monitored to detect attack patterns. When an attack is detected, the firewall policies will be updated on the stateful inspection mechanism. Thus, all the packets to and from the network elements and their corresponding end devices are continuously monitored to secure the entire network from security threats.

## 5. Results and Discussion

A Spyder machine learning tool is used to assess the accuracy of the used ML models. Honeypot attack data from “AWS” was used for training purposes. The honeypot from Amazon web services was a trap spot that can be integrated into the system to identify incoming attempts from data pullers and malicious bots. If a source accesses the honeypot, the IP addresses will be registered. AWS honeypot attack dataset also includes analysis of the data by capturing used IP addresses, profiles, logins, and analyzing resemblance and the total number of instances utilized attack patterns. As of now, the information is being collected using nine distinct honeypots. A comprehensive overview of the data is given in Table 2. Three distinct datasets were being used. Datasets 1 comprised whole data, dataset 2 did not comprise China’s attack data and dataset 3 featured only China’s attack data. ML algorithms were trained using these three datasets and, from there, tested them to detect possible susceptible hosts. For training and testing purposes, the datasets were divided into 30/70, 40/60, 50/50, 60/40, and 70/30 ratios. The threshold values were changed to display the predictive accuracy of various ML methods for distinct datasets. The maximum of 99.98 percent accuracy was achieved of Dataset 1 using the Decision Table algorithm with 0%. It is obvious that the prediction accuracy was considerably affected by the selection of threshold value ubiquity, the machine learning algorithms, and the train/test split ratio.

### 5.1. The Threshold Effect on Predictability

The result findings give a picture that the threshold value has a drastic impact on the host’s predictive accuracy. It is obvious that the rise in the value of the threshold reportedly impacts the accuracy of the prediction. The threshold range rise from 0–10% decreased the accuracy of the prediction by 22.09%. The outcome was noticeable as it suggests that it should be considered serious and not overlook even the smallest attack probability on a specific host. The attacker IP and Port Socket should be denied, and the firewall rules should be updated to suit vulnerable hosts to secure the network.

**Figure 8:**
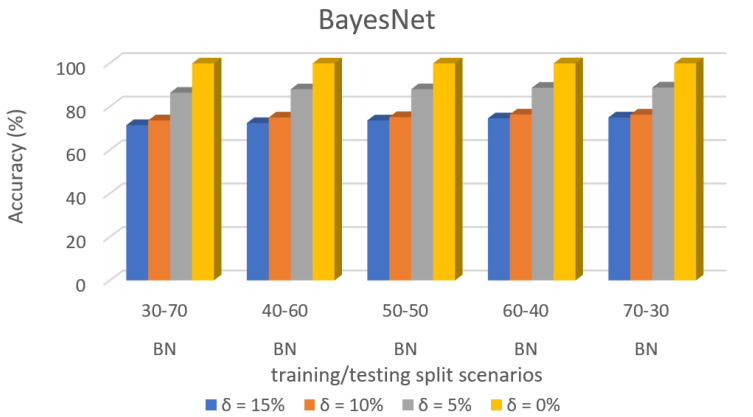
Accuracy Prediction for Dataset 1 using Bayesian Network Algorithm.

Predictive accuracy of specific dataset 3 in ML algorithms with separate training/test split cases and threshold (δ). Figure 8 shows the accuracy prediction for dataset 1 using the Bayesian network algorithm. The result has accuracy % in the y-axis and training/testing split scenarios in the x-axis. The training/testing split scenarios was varied as 30–70, 40–60, 50–50, 60–40, and 70–30 for different threshold (δ) values such as δ = 0%, 5%, 10%, 15%. Thus, the threshold value δ varied the accuracy percentage of the bayesnet algorithm for training/testing split scenario 30/70 were (99.85, 86.36, 73.65, and 71.45)%. The accuracy percentage for training/testing split scenario 40/60 were (99.86, 87.94, 74.98, and 72.38)%. The accuracy percentage for training/testing split scenario 50/50 were (99.86, 87.95, 75.13, and 73.62)%. The accuracy percentage for training/testing split scenario 60/40 were (99.88, 88.63, 76.35, and 74.57)%. The accuracy percentage for training/testing split scenario 70/30 were (99.88, 88.76, 76.29, and 74.98)%. When the threshold value keeps on increasing, the accuracy percentage of the bayesnet algorithm goes down for respective threshold values. The result showed that the prediction percentage of training/test split ratio 70/30 was high for all the respective threshold (δ) values.

**Figure 9:**
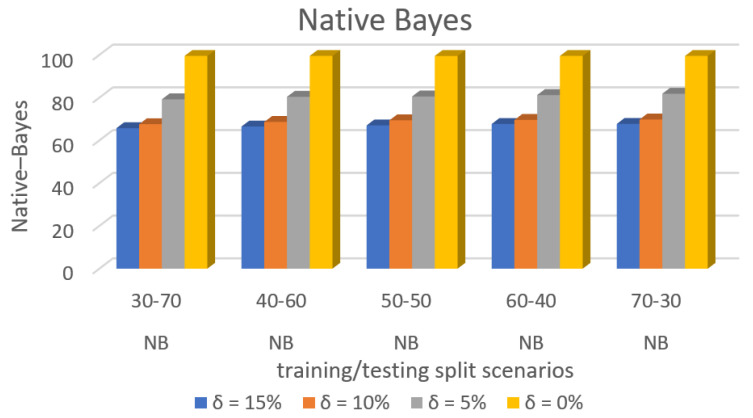
Accuracy Prediction for Dataset 1 using Native Bayes Algorithm.

Figure 9 shows the accuracy prediction for dataset 1 using the Native–Bayes algorithm. The result has accuracy % in the y-axis and training/testing split scenarios in the x-axis. The training/testing split scenarios was varied as 30–70, 40–60,50–50, 60–40, and 70–30 for different threshold (δ) values such as δ = 0%, 5%, 10%, 15%. Thus, the threshold value δ varied the accuracy percentage of the Native–Bayes algorithm for training/testing split scenario 30/70 were (99.53, 79.23, 67.60, and 65.72)%. The accuracy percentage for training/testing split scenario 40/60 were (99.53, 80.42, 68.65, and 66.51)%. The accuracy percentage for training/testing split scenario 50/50 were (99. 52, 80.56, 69.40, and 67.02)%. The accuracy percentage for training/testing split scenario 60/40 were (99.53, 81.23, 69.59, and 67.69)%. The accuracy percentage for training/testing split scenario 70/30 were (99.53, 81.84, 69.79, and 67.74)%. When the threshold value keeps on increasing, the accuracy percentage of the Native–Bayes algorithm goes down for respective threshold values. The result showed that the prediction percentage of training/test split ratio 70/30 was high for all the respective threshold (δ) values.

**Figure 10:**
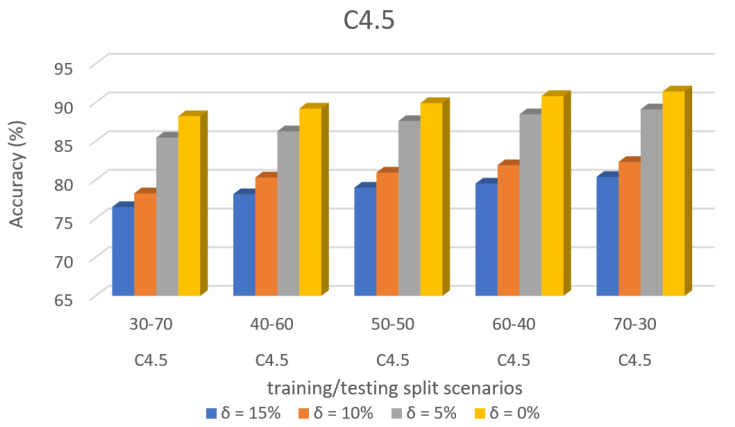
Accuracy prediction for Dataset 1 using the C4.5 algorithm.

Figure 10 shows the accuracy prediction for dataset 1 using the C4.5 algorithm. The result has accuracy % in the y-axis and training/testing split scenarios in the x-axis. The training/testing split scenarios was varied as 30–70, 40–60,50–50, 60–40, and 70–30 for different threshold (δ) values such as δ = 0%, 5%, 10%, 15%. Thus, the threshold value δ varied the accuracy percentage of the C4.5 algorithm for training/testing split scenario 30/70 were (88.23, 85.45, 78.26, and 76.49)%. The accuracy percentage for training/testing split scenario 40/60 were (89.21, 87.60, 80.94, and 78.99)%. The accuracy percentage for training/testing split scenario 50/50 were (89.92, 87.60, 80.94, and 78.99)%. The accuracy percentage for training/testing split scenario 60/40 were (90.83, 88.48, 81.89, and 79.51)%. The accuracy percentage for training/testing split scenario 70/30 were (91.42, 89.11, 82.31, and 80.38)%. When the threshold value keeps on increasing, the accuracy percentage of the C4.5 algorithm goes down for respective threshold values. The result shows that the prediction percentage of training/test split ratio 70/30 was high for all the respective threshold (δ) values.

**Figure 11:**
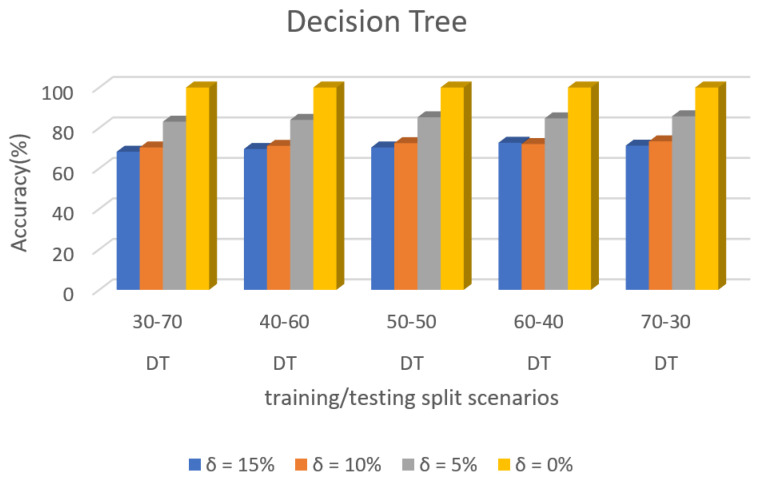
Accuracy Prediction for Dataset 1 using Decision Tree Algorithm.

Figure 11 shows the accuracy prediction for dataset 1 using Decision Tree algorithm. The result has accuracy % in the y-axis and training/testing split scenarios in the x-axis. The training/testing split scenarios was varied as 30–70, 40–60,50–50, 60–40, and 70–30 for different threshold (δ) values such as δ = 0%, 5%, 10%, 15%. Thus, the threshold value δ varied the accuracy percentage of Decision Tree algorithm for training/testing split scenario 30/70 were (99.98, 83.09, 70.35, and 68.21)%. The accuracy percentage for training/testing split scenario 40/60 were (99.98, 83.97, 71.16 and 69.59)%. The accuracy percentage for training/testing split scenario 50/50 were (99.98, 85.25, 72.49, and 70.37)%. The accuracy percentage for training/testing split scenario 60/40 were (99.98, 84.74, 72.05, and 72.68)%. The accuracy percentage for training/testing split scenario 70/30 were (99.98, 85.77, 73.36, and 71.26)%. When the threshold value keeps on increasing, the accuracy percentage of the Decision Tree algorithm goes down for respective threshold values. The result showed that the prediction percentage of training/test split ratio 70/30 was high for all the respective threshold (δ) values.

### 5.2. The ML Method Effect

Figure 8, Figure 9, Figure 10, Figure 11, Figure 12, Figure 13, Figure 14, Figure 15, Figure 16, Figure 17, Figure 18 and Figure 19 provide a perspective into the impact of the ML method on predictive accuracy, and it can be seen that BayesNet achieves the largest overall predictive accuracy of 92.87%.

### 5.3. The Train/Test Split Ratio Effect

The divided train/test ratio also impacts the precision of the prediction. However, with the complexity of the data collected from the “AWS honeypot attack dataset”, the variation in the training/testing split proportion did not significantly alter the predictive accuracy for a particular Threshold value.

Predictive accuracy of specific dataset 2 in ML algorithms with separate training/test split cases and threshold (δ).

**Figure 12:**
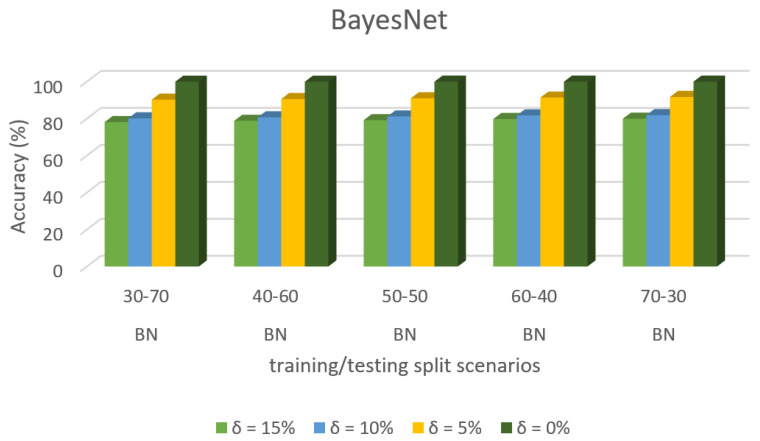
Accuracy Prediction for Dataset 2 using Bayesian Network Algorithm.

Figure 12 shows the accuracy prediction for dataset 2 using the bayesian network algorithm. The result has accuracy % in the y-axis and training/testing split scenarios in the x-axis. The training/testing split scenarios was varied as 30–70, 40–60,50–50, 60–40, and 70–30 for different threshold (δ) values such as δ = 0%, 5%, 10%, 15%. Thus, the threshold value δ varied the accuracy percentage of the bayesnet algorithm for training/testing split scenario 30/70 were (99.92, 90.22, 80.09, and 78.08)%. The accuracy percentage for training/testing split scenario 40/60 were (99.92, 90.53, 80.65, and 78.76)%. The accuracy percentage for training/testing split scenario 50/50 were (99.93, 90.96, 81.12, and 7.13)%. The accuracy percentage for training/testing split scenario 60/40 were (99.94, 91.31, 81.71, and 79.74)%. The accuracy percentage for training/testing split scenario 70/30 were (99.94, 91.68, 81.83, and 79.89)%. When the threshold value increases, the accuracy percentage of the bayesnet algorithm goes down for respective threshold values. The result shows that the prediction percentage of training/test split ratio 70/30 was high for all the respective threshold (δ) values.

**Figure 13:**
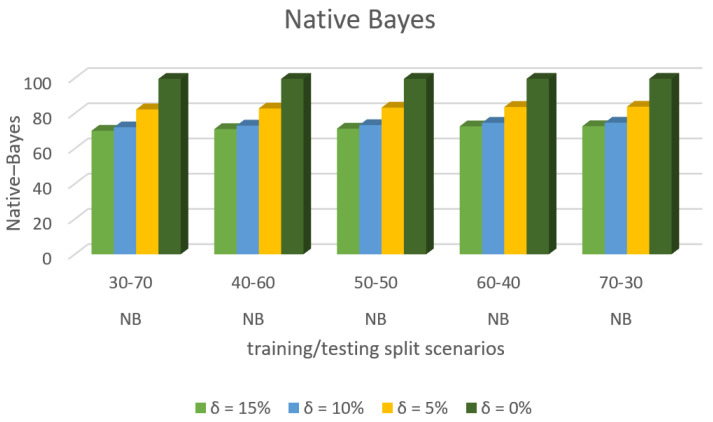
Accuracy Prediction for Dataset 2 using Native–Bayes Algorithm.

Figure 13 shows the accuracy prediction for dataset 2 using the Native–Bayes algorithm. The result had accuracy % in the y-axis and training/testing split scenarios in the x-axis. The training/testing split scenarios was varied as 30–70, 40–60,50–50, 60–40, and 70–30 for different threshold (δ) values such as δ = 0%, 5%, 10%, 15%. Thus, the threshold value δ varies the accuracy percentage of the Native–Bayes algorithm for training/testing split scenario 30/70 were (99.60, 82.27, 72.11, and 70.14)%. The accuracy percentage for training/testing split scenario 40/60 were (99.61, 82.81, 73.07, and 71.09)%. The accuracy percentage for training/testing split scenario 50/50 were (99.62, 83.27, 73.35, and 71.33)%. The accuracy percentage for training/testing split scenario 60/40 were (99.62, 83.66, 74.66, and 72.64)%. The accuracy percentage for training/testing split scenario 70/30 were (99.64, 83.79, 74.74, and 72.73)%. When the threshold value keeps on increasing, the accuracy percentage of the Native–Bayes algorithm goes down for respective threshold values. The result shows that the prediction percentage of training/test split ratio 70/30 was high for all the respective threshold (δ) values.

**Figure 14:**
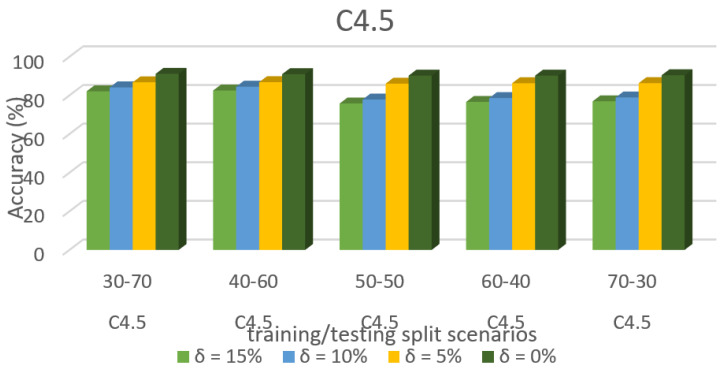
Accuracy Prediction for Dataset 2 using the C4.5 Algorithm.

Figure 14 shows the accuracy prediction for dataset 2 using the C4.5 algorithm. The result has accuracy % in the y-axis and training/testing split scenarios in the x-axis. The training/testing split scenarios was varied as 30–70, 40–60,50–50, 60–40, and 70–30 for different threshold (δ) values such as δ = 0%, 5%, 10%, 15%. Thus, the threshold value δ varies the accuracy percentage of the C4.5 algorithm for training/testing split scenario 30/70 were (91.17, 86.78, 84.17, and 82.13)%. The accuracy percentage for training/testing split scenario 40/60 were (90.99, 86.86, 77.87, and 75.83)%. The accuracy percentage for training/testing split scenario 50/50 were (90.24, 86.29, 78.63, and 76.64)%. The accuracy percentage for training/testing split scenario 60/40 were (90.24, 86.29, 78.63, and 76.64)%. The accuracy percentage for training/testing split scenario 70/30 were (90.44, 86.34, 78.93, and 76.94)%. When the threshold value keeps on increasing, the accuracy percentage of the C4.5 algorithm goes down for respective threshold values. The result showed that the prediction percentage of training/test split ratio 70/30 was high for all the respective threshold (δ) values.

**Figure 15:**
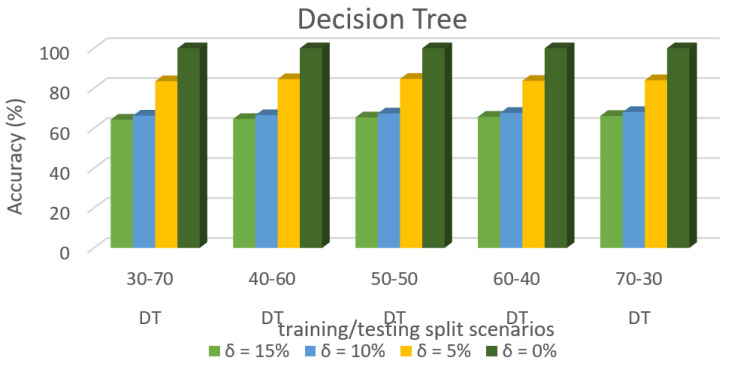
Accuracy Prediction for Dataset 2 using Decision Tree Algorithm.

Figure 15 shows the accuracy prediction for dataset 2 using the Decision Tree algorithm. The result has accuracy % in the y-axis and training/testing split scenarios in the x-axis. The training/testing split scenarios was varied as 30–70, 40–60,50–50, 60–40, and 70–30 for different threshold (δ) values such as δ = 0%, 5%, 10%, 15%. Thus, the threshold value δ varies the accuracy percentage of Decision Tree algorithm for training/testing split scenario 30/70 were (99.98, 83.48, 66.26, and 64.24)%. The accuracy percentage for training/testing split scenario 40/60 were (99.98, 84.51, 66.50, and 64.57)%. The accuracy percentage for training/testing split scenario 50/50 were (99.98, 84.64, 67.35, and 65.37)%. The accuracy percentage for training/testing split scenario 60/40 were (99.98, 83.68, 67,61, and 65.67)%. The accuracy percentage for training/testing split scenario 70/30 were (99.98, 83.98, 68.06, and 66.12)%. When the threshold value keeps on increasing, the accuracy percentage of the Decision Tree algorithm goes down for respective threshold values. The result showed that the prediction percentage of training/test split ratio 70/30 was high for all the respective threshold (δ) values.

### 5.4. The Dataset Effects

The dataset also performs a major part in the accuracy of prediction. The greater the data variability, the greater the likelihood of incorrect prediction will be. Since dataset 2 did not have the Chinese attacker’s data points, the data set pattern was significantly lower than data set 1 and data set 3. This is why the overall prediction accuracy for dataset 2 is greater than for datasets 1 and 3.

Predictive accuracy of specific dataset 3 in ML algorithms with separate training/test split cases and threshold (δ).

**Figure 16:**
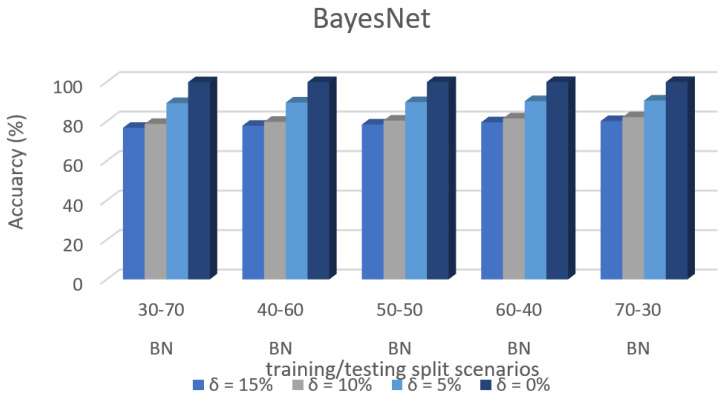
Accuracy Prediction for Dataset 3 using Bayesian Network Algorithm.

Figure 16 shows the accuracy prediction for dataset 3 using the Bayesian network algorithm. The result has accuracy % in the y-axis and training/testing split scenarios in the x-axis. The training/testing split scenarios was varied as 30–70, 40–60,50–50, 60–40, and 70–30 for different threshold (δ) values such as δ = 0%, 5%, 10%, 15%. Thus, the threshold value δ varied the accuracy percentage of the bayesnet algorithm for training/testing split scenario 30/70 were (99.83, 89.27, 78.73, and 76.76)%. The accuracy percentage for training/testing split scenario 40/60 were (99.88, 89.56, 79.77, and 77.78)%. The accuracy percentage for training/testing split scenario 50/50 are (99.92, 89.77, 80.42, and 78.43)%. The accuracy percentage for training/testing split scenario 60/40 were (99.93, 90.26, 81.51, and 79.54)%. The accuracy percentage for training/testing split scenario 70/30 were (99.93, 90.55, 82.14, and 80.18)%. When the threshold value keeps on increasing, the accuracy percentage of the bayesnet algorithm goes down for respective threshold values. The result shows that the prediction percentage of training/test split ratio 70/30 was high for all the respective threshold (δ) values.

**Figure 17:**
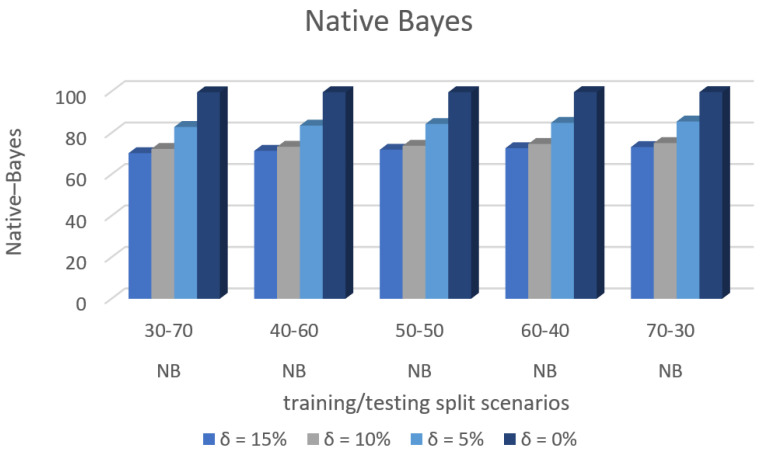
Accuracy Prediction for Dataset 3 using Native–Bayes Algorithm.

Figure 17 shows the accuracy prediction for dataset 3 using the Native–Bayes algorithm. The result has accuracy % in the y-axis and training/testing split scenarios in the x-axis. The training/testing split scenarios was varied as 30–70, 40–60,50–50, 60–40, and 70–30 for different threshold (δ) values such as δ = 0%, 5%, 10%, 15%. Thus, the threshold value δ varied the accuracy percentage of the Native–Bayes algorithm for training/testing split scenario 30/70 were (99.60, 82.87, 72.27, and 70.22)%. The accuracy percentage for training/testing split scenario 40/60 were (99.62, 83.51, 73.31, and 71.34)%. The accuracy percentage for training/testing split scenario 50/50 were (99.65, 84.30, 73.78, and 71.89)%. The accuracy percentage for training/testing split scenario 60/40 were (99.67, 84.86, 74.68, and 72.63)%. The accuracy percentage for training/testing split scenario 70/30 were (99.68, 85.48, 75.10 and 73.17)%. When the threshold value keeps on increasing, the accuracy percentage of Native–Bayes algorithm goes down for respective threshold values. The result showed that the prediction percentage of training/test split ratio 70/30 was high for all the respective threshold (δ) values.

**Figure 18:**
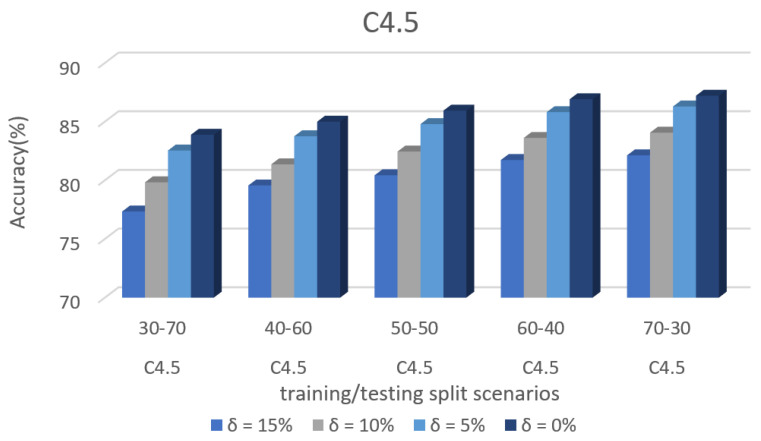
Accuracy Prediction for Dataset 3 using the C4.5 Algorithm.

Figure 18 shows the accuracy prediction for dataset 3 using the C4.5 algorithm. The result has accuracy % in the y-axis and training/testing split scenarios in the x-axis. The training/testing split scenarios was varied as 30–70, 40–60,50–50, 60–40, and 70–30 for different threshold (δ) values such as δ = 0%, 5%, 10%, 15%. Thus, the threshold value δ varied the accuracy percentage of C4.5 algorithm for training/testing split scenario 30/70 were (83.88, 82.54, 79.83, and 77.34)%. The accuracy percentage for training/testing split scenario 40/60 were (85, 83.75, 81.36, and 79.56)%. The accuracy percentage for training/testing split scenario 50/50 were (85.94, 84.79, 82.45, and 80.43)%. The accuracy percentage for training/testing split scenario 60/40 were (86.91, 85.81, 83.61, and 81.73)%. The accuracy percentage for training/testing split scenario 70/30 were (87.21, 86.29, 84.06, and 82.13)%. When the threshold value keeps on increasing, the accuracy percentage of the C4.5 algorithm goes down for respective threshold values. The result showed that the prediction percentage of training/test split ratio 70/30 was high for all the respective threshold (δ) values.

**Figure 19:**
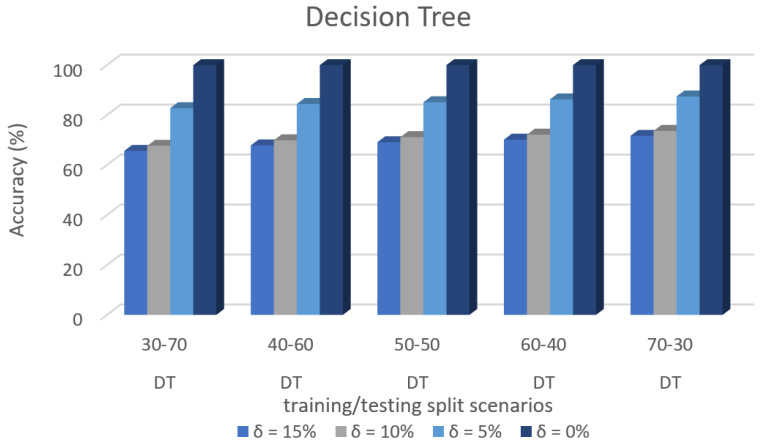
Accuracy Prediction for Dataset 3 using Decision Tree Algorithm.

Figure 19 shows the accuracy prediction for dataset 3 using the Decision Tree algorithm. The result has accuracy % in the y-axis and training/testing split scenarios in the x-axis. The training/testing split scenarios is varied as 30–70, 40–60,50–50, 60–40, and 70–30 for different threshold (δ) values such as δ = 0%, 5%, 10%, 15%. Thus, the threshold value δ varies the accuracy percentage of the Decision Tree algorithm for training/testing split scenario 30/70 are (99.98, 82.76, 67.69, and 65.64)%. The accuracy percentage for training/testing split scenario 40/60 are (99.98, 84.44, 69.89, and 67.83)%. The accuracy percentage for training/testing split scenario 50/50 are (99.98, 85.12, 71.16, and 69.12)%. The accuracy percentage for training/testing split scenario 60/40 are (99.98, 86.28, 72.16, and 70.14)%. The accuracy percentage for training/testing split scenario 70/30 are (99.98, 87.43, 73.65, and 71.67)%. When the threshold value keeps on increasing, the accuracy percentage of the Decision Tree algorithm goes down for respective threshold values. The result shows that the prediction percentage of training/test split ratio 70/30 was high for all the respective threshold (δ) values.

The results in Figure 8, Figure 9, Figure 10, Figure 11, Figure 12, Figure 13, Figure 14, Figure 15, Figure 16, Figure 17, Figure 18 and Figure 19 showed that the susceptible host can be predicted correctly by ML methods. Then, SDN Moule can leverage such accurate prediction to prevent the future attacker from entering the network and assist in protecting various hosts from DOS attacks.

## 6. Conclusions

The machine learning method is used in this paper to predict the susceptible host that is extremely probable to be assaulted in the SDNFV network with distributed controllers. Using the prediction results of machine learning models, it is possible to define the security flow rules for stateful firewall VNF to avoid unauthorized clients from entering the network. Experimental findings have shown that machine learning methods can help define stateful firewall security flow rules by anticipating the potential susceptible host correctly to deny DOS network attacks. Stateful inspection firewalls have been regarded to be safer than stateless firewalls. Therefore, they are able to take a deeper look into the transaction to understand the network operations. If it is vital to complete a transaction, ports are dynamically opened and closed. AWS honeypot attack dataset is used to train and test the proposed system using four machine learning algorithms, Bayesian Network, Native–Bayes Algorithm, C4.5, and Decision Tree algorithm to predict possible network attacks. Bayesian Network algorithm achieved an average prediction accuracy of 92.87%, Native–Bayes Algorithm achieved an average prediction accuracy of 87.81%, C4.5 Algorithm achieved an average prediction accuracy of 84.92%, and Decision Tree algorithm achieved an average prediction accuracy of 83.18%. Indicating that out of the total of 450 k attacks, the Bayesian network was able to identify 419 k attacks correctly. Furthermore, the decrease in prediction accuracy with the threshold rise stated that the smallest chance of attack threat is not to be ignored, and the firewall security policies on the stateful firewall VNF module should be altered to deny the potential risk.

## Figures and Tables

**Table 1:** Hardware and software configuration and version.

Components	Description and Version
CPU	Intel core i5-8250U @ 1.60Ghz
Memory	8 GB
OS	Windows 10 home edition
hypervisor	Oracle VirtualBox-5.1.30
VM OS	Ubuntu 14.04 (64 bit)
VM configuration	1 CPU core, 1 GB memory
Mininet emulator	2.2.1
POX controller	0.2.0 (carp)
Coding	Python 2.7
Communication Protocol	OpenFlow 1.3

**Table 2:** Shows the descriptions about the dataset.

Data Set	Size	Format
**1**	451518 (Overall attack data including China)	[attacker IP] [attacker Port] [attacked host] [number of attack attempts] [time stamp]
**2**	260124 (attack data excluding China)	[attacker IP] [attacker Port] [attacked host] [number of attack attempts] [time stamp]
**3**	191394 (attack data only from China)	[attacker IP] [attacker Port] [attacked host] [number of attack attempts] [time stamp]

## Data Availability

Not Applicable.
